# Epidemiological and clinical features of SARS-CoV-2 Omicron variant infection in Quanzhou, Fujian province: a retrospective study

**DOI:** 10.1038/s41598-023-49098-x

**Published:** 2023-12-13

**Authors:** Huatang Zhang, Zhangyan Weng, Yijuan Zheng, Minghui Zheng, Wenhuang Chen, Haoyi He, Xiaoyi Ye, Youxian Zheng, Jianfeng Xie, Kuicheng Zheng, Jiming Zhang, Xibin Zhuang, Zhijun Su, Yongjun Zhou, Xueping Yu

**Affiliations:** 1 Department of Infection Disease, Fujian Medical University Affiliated First Quanzhou Hospital, No. 250 East Street, Licheng District, Quanzhou, 362000 Fujian China; 2 Department of Infection Disease, Quanzhou Guangqian Hospital, Guangqian South Street, Nan’an, Quanzhou, 362000 Fujian China; 3 Department of Respiratory Disease, Fujian Medical University Affiliated First Quanzhou Hospital, No. 250 East Street, Licheng District, Quanzhou, 362000 Fujian China; 4 Department of Clinical Laboratory, Quanzhou Center for Disease Control and Prevention, No. 21 Jinhuai Street, Fengze District, Quanzhou, 362000 Fujian China; 5 Fujian Provincial Key Laboratory of Zoonosis Research, Fujian Center for Disease Control and Prevention, No. 76 Jintai Road, Gulou District, Fuzhou, 350001 Fujian China; 6 Department of Infection Disease, Huashan Hospital Fudan University, No. 12 Wulumuqi Middle Road, Shanghai, 200040 China; 7 Institute of Bioengineering and Biotechnology, College of Life Sciences and Chemistry, Minnan Science and Technology University, Quanzhou, 362000 China

**Keywords:** Biochemistry, Cell biology, Immunology, Microbiology, Biomarkers, Diseases, Gastroenterology, Health occupations, Medical research, Risk factors, Signs and symptoms

## Abstract

Epidemiological and clinical data of patients infected with severe acute respiratory syndrome coronavirus 2 (SARS-CoV-2) Omicron variant (BA.2) admitted to three designated hospitals in Quanzhou City, Fujian Province, China, were collected and analyzed. Overall, 2,541 patients infected with BA.2, comprising 1,060 asymptomatic, 1,287 mild, and 194 moderate infections, were enrolled. The percentage of moderate infections was higher in patients aged ≥ 60 years than in those aged < 18 years and 18–59 years. The median hospitalization duration was 17 days. Among the 2,541 patients, 43.52% had a clear history of close contact. The vaccination rate was 87.92%, and the percentage of asymptomatic infections was higher in vaccinated than in unvaccinated patients. Moreover, patients with underlying diseases, including hypertension and diabetes mellitus, had more moderate infections than those without underlying diseases. The three most common clinical manifestations were fever, dry cough, and sore throat. The albumin-to-globulin (A/G) ratio and lymphocyte count decreased in cases with mild and moderate infections, while procalcitonin, erythrocyte sedimentation rate, interleukin-6, D-dimer, and C4 levels increased. Advanced age, non-vaccination, and underlying comorbid diseases were high-risk factors for disease progression in patients. However, dynamic monitoring of blood routine parameters, A/G ratio, and inflammatory indicators facilitated the prediction of disease progression.

## Introduction

Severe acute respiratory syndrome coronavirus 2 (SARS-CoV-2) is highly contagious and can easily create modes of avoidance to evade immune system attacks. It continuously mutates and evolves, producing mutant strains, such as Alpha (B.1.1.7), Beta (B.1.351), Gamma (P.1), Delta (B.1.617.2), and Omicron (B.1.1.529), with varying transmissibility, pathogenicity, and immune escape ability^1^. The World Health Organization received the first report of an infection with the SARS-CoV-2 Omicron variant in South Africa on November 24, 2021, and upgraded its classification to concern variants on November 26 of the same year^2^. Omicron variants have since spread rapidly to many countries and regions worldwide and are now the dominant strains, threatening human health and life. The mutants have high transmissibility with a shorter generation time and incubation period, as well as a stronger ability to evade the immune system. The effectiveness of the coronavirus disease 2019 (COVID-19) vaccines in preventing infection, illness, hospitalization, and death diminishes over time, with mutations in the spike (S) protein and resulting mutants posing new challenges to the development and application of these vaccines, as well as to prevention and control of the COVID-19 pandemic^3^. COVID-19 vaccines may cause side effects in some individuals, including rare abnormal blood clotting and heart inflammation^4^. COVID-19 can affect the entire range of infected individuals, in whom multiple organs and systems may be involved and affected, including but not limited to the respiratory system, cardiovascular system, nervous system, gastrointestinal system, and musculoskeletal system. The “post-acute sequelae of SARS-CoV-2 infection (PASC)” is highly heterogeneous, with unclear etiology and different pathogenesis, which requires further clarification^5^.

To our knowledge, there are few reports about real-world studies on COVID-19 in China, especially in the southeast coastal areas. Existing reports have included a few COVID-19 cases and evaluation indicators, which cannot comprehensively and objectively describe the clinical characteristics of the population under the state of natural COVID-19. In March 2022, a sudden outbreak of COVID-19 occurred in Quanzhou City, Fujian Province, China. Nucleic acid testing of clinical samples by the Quanzhou Center for Disease Control and Prevention (CDC) showed that the strain was a SARS-CoV-2 Omicron sublineage (BA.2). After comparing it with the current SARS-CoV-2 sequence database in China, the Chinese CDC confirmed that the mutant strain was newly discovered in China at that time. The newly emerged recombinant coronavirus variant may have the characteristics of high transmissibility of SARS-CoV-2, which may have a catastrophic impact. Therefore, there is an urgent need to develop broad-spectrum vaccines and therapeutics that are effective against beta-coronavirus in order to prevent possible future outbreaks of SARS-CoV-2^6^. Few retrospective clinical studies on the SARS-CoV-2 Omicron variant have been reported in China. Therefore, this study analyzed the epidemiological and clinical features of BA.2 infections that caused the current epidemic outbreak in Quanzhou, aiming to provide a scientific basis for clinical diagnosis and treatment of the disease and accurate prognostic prediction.

## Methods

### Ethics declarations

Informed consent was obtained from all patients and/or their legal guardians. This study was approved by the Ethics Committee of the First Hospital of Quanzhou, Affiliated with Fujian Medical University (Quan Yi Lun 2023; approval number: K004). The study was conducted in accordance with the relevant guidelines and regulations of the Chinese Health Commission on the prevention and control of COVID-19.

### Source of cases

This study enrolled patients who had either asymptomatic infections or confirmed symptomatic infections of Omicron variant strain BA.2 and were admitted between March 13, 2022, and April 14, 2022, at the Chengdong Branch and Infectious Diseases Branch of Fujian Medical University Affiliated First Quanzhou Hospital, as well as the Quanzhou "Huoweishan" Cabin Hospital. Patients suspected of recovering from COVID-19 but re-tested positive for SARS-CoV-2, with previous infection of the novel coronavirus, originating from regions other than Quanzhou, and with significant missing information on medical records were excluded from this study.

### Diagnostic criteria

Patients with an epidemiological history and clinical manifestation of novel coronavirus infections along with serological or pathogenic evidence were defined as having a confirmed diagnosis of COVID-19 according to the COVID-19 Diagnosis and Treatment Protocol (Trial Version 9)^7^ issued by the Office of the National Health Care Commission and Office of the State Administration of Traditional Chinese Medicine. According to clinical manifestations and auxiliary examinations, confirmed cases were classified as mild, moderate, severe, and critical. Asymptomatic infections were defined as testing positive for the novel coronavirus and having no associated clinical manifestations according to the Prevention and Control Plan for Novel Coronavirus Disease (Version 8) issued by the Integrated Group of the State Council for Joint Prevention and Control of Novel Coronavirus Disease Epidemic^8^.

### Data collection

Information on demographic characteristics, such as sex, age, body mass index, underlying disease, and vaccination status for COVID-19, as well as disease classification and clinical manifestations, was obtained. Auxiliary examinations, including routine tests for blood parameters, inflammatory markers, humoral immunity, T-lymphocyte subsets (absolute counts), coagulation, complete biochemical parameters, and COVID-19 antibodies; quantitative reverse-transcription polymerase chain reaction (RT-qPCR) cycle threshold (CT) for nucleic acid testing; pulmonary imaging (except in pregnant women); and post-treatment testing, were performed within 48 h of admission. Moreover, each case was evaluated as to whether Western or traditional Chinese medical treatments were to apply. The efficacy was evaluated by recording the time interval from testing positive to testing negative for SARS-CoV-2. Strict regulations of database management were implemented to ensure data security and confidentiality.

### Statistical methods

All statistical analyses were performed using SPSS 23.0 (IBM Corp., Armonk, NY, USA). Measurement data were analyzed using the Kruskal–Wallis rank-sum or Mann–Whitney U tests and expressed as medians (25th and 75th percentiles). Count data were subjected to the χ2 or Fisher’s exact test and expressed as percentages. Differences with a *P*-value of < 0.05 were considered statistically significant.

## Results

### Epidemiological, demographic, and clinical features

**Figure 1:**
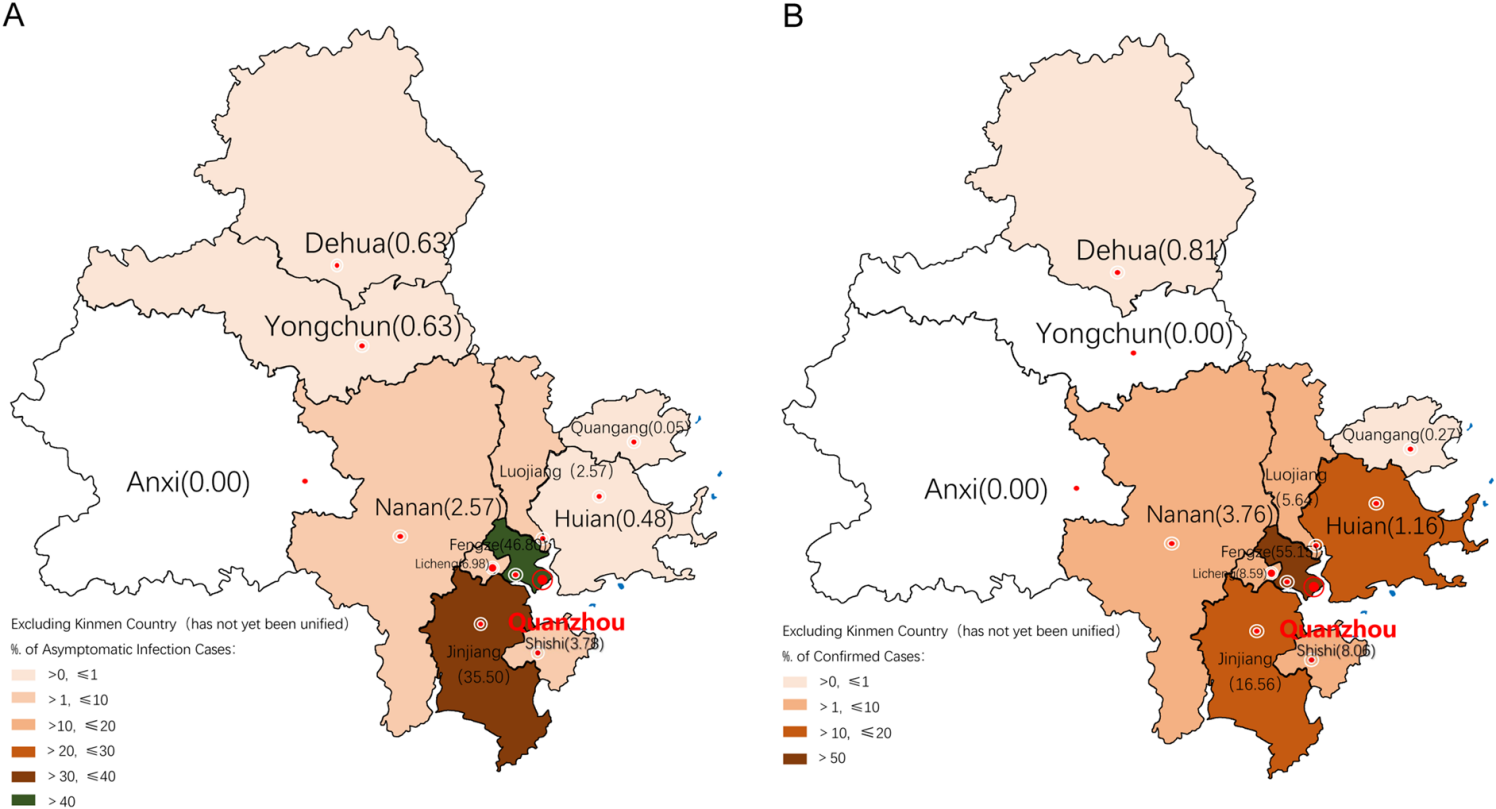
Distribution of patients with coronavirus disease 2019 (COVID-19) across Quanzhou City. **(AB)** Fengze District had the highest number of asymptomatic (**A**) and confirmed cases (**B**), followed by Jinjiang City, Licheng District, Shishi City, Luojiang District, Nan'an City, Hui'an County, Dehua County, and Quangang District. The map in Fig. 1 was generated using MS Paint. Version number: Windows 10 Home Chinese Version 21H2.URL link: https://www.microsoft.com/zh-cn/software-download/windows10.

A total of 2,541 patients infected with SARS-CoV-2 Omicron sublineage (BA.2) were enrolled in this study, of whom 1,060 (41.72%) were asymptomatic, and 1,481 (58.28%) had a confirmed diagnosis of COVID-19. Of the confirmed cases, 1,287 (86.90%) were mild and 194 (13.10%) were moderate, but there were no severe or critical cases. Of the 2,541 patients, 1,106 (43.52%), 901 (35.47%), and 34 (21.01%) had a confirmed, uncertain, and no history of contact, respectively. Regarding the number of infected patients (whether asymptomatic or confirmed cases), most patients were from Fengze District (46.80% and 55.15%), followed by Jinjiang City, Licheng District, Shishi City, Luojiang District, Nan’an City, Hui’an County, Dehua County, and Quangang District. Meanwhile, no confirmed cases were reported in Yongchun County, and no Omicron variant infections were reported in Anxi County (Fig. 1). The percentages of patients who received the one, two, and three doses of the COVID-19 vaccine were 5.16%, 41.95%, and 40.82%, respectively, while that of unvaccinated patients was 10.19%. The percentage of asymptomatic infections was higher while the percentage of mild infections was lower in vaccinated than in unvaccinated patients (42.75% vs. 36.29%, χ^2^ = 3.97, *P* = 0.046; and 49.69% vs. 56.76%, χ^2^ = 4.64, *P* = 0.031; respectively). However, there was no significant difference in the percentage of moderate infections (7.56% vs. 6.95%, *P* = 0.72) between the vaccinated and unvaccinated groups, with no association of the between-group difference or similarity with the number of doses of the COVID-19 vaccine.

As shown in Table 1, there were 1,218 male patients (47.93%), of whom 51.04% (541) were asymptomatic, which was higher than those with moderate infections (41.75%, *n* = 81). The age of the patients ranged from 1 to 97 years, with a median of 35 (24–48) years. Moreover, the infected patients were mainly aged between 18 and 59 years (76.34%). Patients aged < 18 years (15.03%) mainly had mild infections (57.33%), which was relatively higher than those aged 18–59 and > 60 years. The percentages of asymptomatic (49.32%) and moderate infections (10.96%) were higher in patients aged ≥ 60 years (8.62%) than in those belonging to the other two age groups (< 18 and 18–39). The median hospitalization duration was 17 (14–20) days, with the longest being 19 days for moderate infections, 17 days for mild infections, and 16 days for asymptomatic infections. A total of 275 patients (10.82%) had at least one underlying comorbid disease, with a high percentage of cases of hypertension (151 cases for 5.94%) and diabetes mellitus (81 cases for 3.12%), followed by coronary heart disease, malignancy, chronic liver disease, thyroid disease, connective tissue disease, and respiratory disease. The percentage of moderate infections was higher in patients with underlying comorbid disease than in those without (12.00% vs. 7.12%, χ2 = 8.26, *P* = 0.004). The most common clinical manifestations in confirmed cases were fever (27.47%), followed by dry cough (25.19%), sore throat (15.31%), fatigue (12.20%), expectoration (10.82%), myalgia (10.74%), stuffy and runny nose (4.80%), anosmia and ageusia (2.99%), diarrhea (2.56%), shortness of breath (1.02%), conjunctivitis (0.31%), as well as nausea and vomiting (0.19%) (Fig. 1).

**Table 1:** Demographic, epidemiological, and clinical features of patients infected with the SARS-CoV-2 Omicron variant BA.2.

Characteristics	All patients (*n* = 2541)	Asymptomatic patients (*n* = 1060)	Mild cases (*n* = 1287)	Moderate cases (*n* = 194)	χ^2^	*P*-value
Sex, male (%)	1218 (47.93%)	541 (51.04%)	596 (49.31%)	81 (41.75%)	8.423	0.0148
Age, median (IQR), years	35 (24–48)	38 (27–51)	33 (22–45)	39 (27.75–51)	56.89	< 0.0001
Age groups, years						
< 18	382 (15.03%)	140 (36.65%)	219 (57.33%)	23 (6.02%)	45.54	< 0.0001
18–39	1124 (44.23%)	423 (37.63%)	625 (55.60%)	76 (6.76%)		
40–59	816 (32.11%)	389 (47.67%)	356 (43.63%)	71 (8.70%)		
≥ 60	219 (8.62%)	108 (49.32%)	87 (39.73%)	24 (10.96%)		
BMI, Median (IQR) (kg/m^2^)	22.52(19.81–25.39)	23.03 (20.08–25.61)	22.22 (19.27–25.21)	22.86 (20.70–25.64)	14.869	0.001
Length of stay, median (IQR), years	17 (14.0–20.0)	16 (12.75–19)	17 (14–20)	19 (16–22)	66.35	< 0.0001
COVID-19 vaccination status						
Unknown	48 (1.89%)	11 (19.64%)	30 (53.57%)	7 (12.5%)	17.87	0.0222
Unvaccinated	259 (10.19%)	94 (36.29%)	147 (56.76%)	18 (6.95%)		
Partially	131 (5.16%)	56 (42.75%)	67 (51.15%)	8 (6.10%)		
Vaccinated	1066 (41.95%)	433 (40.62%)	550 (51.59%)	83 (7.78%)		
Boosted	1037 (40.82%)	466 (44.94%)	493 (47.54%)	78 (7.52%)		
Underlying disease						
Hypertension	151 (5.94%)	74 (49.01%)	57 (37.7%)	20 (13.25%)	13.939	0.001
Diabetes	81 (3.19%)	34 (42.00%)	43 (53.10%)	4 (4.90%)	0.896	0.634^a^
Coronary heart disease	25 (0.98%)	9 (36.00%)	14 (56.00%)	2 (8.00%)	0.456	0.777^a^
Malignant tumor	20 (0.79%)	9 (45.0%)	7 (35.0%)	4 (20.0%)	4.668	0.069^a^
Chronic liver disease	18 (0.71%)	2 (11.10%)	14 (77.80%)	2 (11.10%)	7.196	0.015^a^
Thyroid disease	15 (0.59%)	3 (20.0%)	9 (60.0%)	3 (20.0%)	4.954	0.074^a^
Connective tissue disease	9 (0.35%)	3 (33.3%)	4 (44.4%)	2 (22.2%)	2.761	0.245^a^
Respiratory disease	7 (0.28%)	4 (57.1%)	2 (28.6%)	1 (14.3%)	2.144	0.312^a^
Signs and symptoms						
Fever	698 (27.47%)	–	622 (89.11%)	76 (10.89%)	5.670	0.0173^a^
Dry cough	640 (25.19%)	–	556 (86.88%)	84 (13.12%)	0.0006	0.9796
Pharyngalgia	389 (15.31%)	–	346 (88.95%)	43 (11.05%)	1.939	0.1638
Fatigue	310 (12.20%)	–	272 (87.74%)	38 (12.26%)	0.2437	0.6215
Expectoration	275 (10.82%)	–	230 (83.64%)	45 (16.36%)	3.161	0.0754
Myalgia	273 (10.74%)	–	243 (89.01%)	30 (10.99%)	1.309	0.2525
Stuffy and runny nose	122 (4.80%)	–	109 (89.34%)	13 (10.66%)	0.6974	0.40375
Anosmia and ageusia	76 (2.99%)	–	66 (86.84%)	10 (13.16%)	0.00027	0.9876^a^
Diarrhea	65 (2.56%)	–	58 (95.5%)	7 (4.5%)	0.390	0.584^a^
Shortness of breath	26 (1.02%)	–	23 (88.46%)	3 (11.54%)	0.05664	1.000^a^
Conjunctivitis	8 (0.31%)	–	7 (87.50%)	1 (12.50%)	0.0021	0.9633^a^
Nausea and vomiting	5 (0.19%)	–	5 (100.0%)	0	0.756	0.3845^a^

^a^Fisher’s exact test.

SARS-CoV-2, severe acute respiratory syndrome coronavirus 2; COVID-19, coronavirus disease 2019; *IQR*, interquartile range; *BMI*, body mass index.

### Results of auxiliary examinations within 48 h of admission

Abnormal alanine transaminase (ALT) levels and hypo-albuminemia were observed in 190 (7.48%) and 312 (12.28%) patients, respectively (Table 2). The lymphocyte count and albumin-to-globulin ratio (A/G) were higher in asymptomatic cases than in mild and moderate cases (all *P* < 0.01), while asymptomatic cases had lower levels of C-reactive protein (CRP), complement C4, interleukin-6 (IL-6), prothrombin, globulin (GLB), and D-dimer than mild and moderate cases (all *P* < 0.05). White blood cell, neutrophil, and platelet counts; total bilirubin (TBIL), ALT, and gamma-glutamyl transferase (GGT) levels; as well as CD3^+^ and CD4^+^ T-cell counts were higher in asymptomatic cases than in mild cases (all *P* < 0.01). Meanwhile, asymptomatic cases had a lower international normalized ratio than mild cases (*P* < 0.001), as well as a lower erythrocyte sedimentation rate (ESR) and immunoglobulin levels than moderate cases (all *P* < 0.01). Blood urea nitrogen levels were higher in asymptomatic cases, and albumin (ALB) levels were higher in mild cases than in moderate cases (both *P* < 0.05). The common imaging findings of moderate cases were patchy opacities (61.34%) and ground-glass opacities (53.09%), followed by strip cord shadow (39.18%) and nodular shadow (14.95%).

**Table 2:** Laboratory and imaging examination results of patients infected with the SARS-CoV-2 Omicron variant BA.2

	All patients (*n* = 2541)	Asymptomatic patients (*n* = 1060)	Mild cases (*n* = 1287)	Moderate cases (*n* = 194)	χ2	*P*
Leucocytes (*10^9^/L)	5.22(4.09–6.65) (*n* = 1981)	5.55(4.36–7.06) (*n* = 684)	5.00(3.93–6.39) (*n* = 1116)	5.28(4.13–6.61) (*n* = 181)	36.054	0.000
Neutrophil (*10^9^/L)	2.82(1.94–4.00) (*n* = 1981)	3.07(2.17–4.17) (*n* = 683)	2.68(1.84–3.87) (*n* = 1117)	2.82(2.08–4.29) (*n* = 181)	21.390	0.000
Lymphocytes (*10^9^/L)	1.19(1.09–2.04) (*n* = 1982)	1.63(1.20–2.21) (*n* = 684)	1.42(1.05–1.92) (*n* = 1117)	1.46(1.01–1.95) (*n* = 181)	38.379	0.000
Neutrophil–to–Lymphocyte ratio	1.77(1.18–2.99) (*n* = 1953)	1.74(1.19–2.80) (*n* = 667)	1.77(1.16–3.06) (*n* = 1105)	1.86(1.28–3.05) (*n* = 181)	1.654	0.437
Monocytes (*10^9^/L)	0.55(0.41–0.72) (*n* = 1980)	0.53(0.41–0.70) (*n* = 683)	0.56(0.42–0.74) (*n* = 1117)	0.55(0.41–.075) (*n* = 180)	4.662	0.097
Platelet (*10^9^/L)	216.00(177.00–261.00) (*n* = 1981)	221.00(182.00–272.00) (*n* = 683)	213.00(173.00–255.00) (*n* = 1117)	213.00(174.05–265.00) (*n* = 181)	12.445	0.002
Red blood cell	4.66(4.31–5.04) (*n* = 1980)	4.67(4.36–5.05) (*n* = 683)	4.66(4.29–5.04) (*n* = 1117)	4.65(4.29–5.04) (*n* = 181)	1.299	0.522
Procalcitonin (ng/ml)	4.30(0.08–6.10) (*n* = 682)	4.30(0.06–5.70) (*n* = 175)	4.40(0.09–6.40) (*n* = 449)	4.50(0.08–5.80) (*n* = 58)	4.421	0.110
C–reactive protein (mg/L)	3.05(0.53–5.68) (*n* = 1956)	2.13(0.51–4.58) (*n* = 661)	3.59(0.55–6.28) (*n* = 1115)	3.90(2.10–7.92) (*n* = 180)	65.119	0.000
Erythrocyte sedimentation rate (mm/H)	15.00(9.00–25.00) (*n* = 1787)	14.00(8.00–23.00) (*n* = 579)	15.00(9.00–25.00) (*n* = 1037)	17.00(10.00–31.00) (*n* = 171)	10.471	0.005
Interleukin–6 (ng/L)	7.59(3.68–76.12) (*n* = 1588)	5.73(2.81–22.57) (*n* = 492)	8.65(4.02–103.75) (*n* = 937)	10.84(4.39–199.10) (*n* = 159)	36.200	0.000
Prothrombin time (S)	11.40(10.90–12.10) (*n* = 1828)	11.30(10.80–11.90) (*n* = 584)	11.50(10.90–12.30) (*n* = 1070)	11.40(10.70–12.10) (*n* = 174)	29.077	0.000
D–dimer (mg/L)	0.26(0.16–0.40) (*n* = 1859)	0.23(0.14–0.36) (*n* = 611)	0.26(0.16–0.43) (*n* = 1073)	0.28(0.19–0.42) (*n* = 175)	23.276	0.000
INR	1.02(0.97–1.07) (*n* = 1826)	1.00(0.96–1.05) (*n* = 611)	1.02(0.97–1.09) (*n* = 1069)	1.01(0.96–1.07) (*n* = 174)	30.781	0.000
Albumin (g/L)	43.20(40.90–45.20) (*n* = 1966)	43.20(40.80–45.20) (*n* = 676)	43.30(41.00–45.30) (*n* = 1109)	42.50(40.25–44.65) (*n* = 181)	7.049	0.029
Globulin (g/L)	26.70(24.20–29.50) (*n* = 1967)	26.50(24.00–29.80) (*n* = 677)	26.60(24.20–29.20) (*n* = 1109)	28.10(25.35–30.40) (*n* = 181)	13.312	0.001
Albumin–to–globulin ratio	1.62(1.15–1.79) (*n* = 1899)	1.63(1.46–1.80) (*n* = 622)	1.63(1.46–1.80) (*n* = 1097)	1.55(1.37–1.71) (*n* = 180)	17.658	0.000
Alanine transaminase (U/L)	17.00(12.00–26.00) (*n* = 1966)	18.00(12.00–28.00) (*n* = 676)	16.00(11.00–25.00) (*n* = 1109)	17.00(12.00–27.00) (*n* = 181)	9.967	0.008
Aspartate aminotransferase (U/L)	23.00(19.00–30.00) (*n* = 1967)	23.00(19.00–29.00) (*n* = 676)	23.00(19.00–30.00) (*n* = 1110)	23.00(19.00–30.00) (*n* = 181)	4.080	0.130
Glutamyl transpeptidase (U/L)	20.00(14.00–35.00) (*n* = 1966)	22.50(15.00–38.75) (*n* = 676)	19.00(14.00–32.50) (*n* = 1109)	21.00(15.00–37.00) (*n* = 181)	16.562	0.000
Alkaline phosphatase (U/L)	70.00(56.00–92.00) (*n* = 1966)	73.00(58.00–92.75) (*n* = 676)	69.00(54.00–92.00) (*n* = 1109)	68.00(56.00–86.50) (*n* = 181)	5.663	0.059
Total bilirubin (μmol/L)	9.20(7.13–12.40) (*n* = 1964)	9.70(7.70–13.50) (*n* = 674)	8.90(6.80–11.80) (*n* = 1109)	8.90(7.30–13.40) (*n* = 181)	29.791	0.000
Blood urea nitrogen (mmol/L)	4.12(3.44–4.98) (*n* = 1937)	4.23(3.49–5.11) (*n* = 651)	4.09(3.43–4.95) (*n* = 1106)	3.89(3.27–4.73) (*n* = 180)	10.056	0.007
Creatinine (μmol/L)	59.20(47.90–74.30) (*n* = 1937)	60.70(49.10–75.80) (*n* = 651)	58.30(47.58–73.80) (*n* = 1106)	58.90(49.63–72.30) (*n* = 180)	3.325	0.190
Complement C3	0.89(0.77–1.01) (*n* = 1507)	0.89(0.77–1.03) (*n* = 475)	0.89(0.76–1.01) (*n* = 877)	0.90(0.80–1.02) (*n* = 155)	1.342	0.511
Complement C4	0.25(0.20–0.30) (*n* = 1508)	0.24(0.19–0.29) (*n* = 476)	0.25(0.21–0.30) (*n* = 877)	0.26(0.22–0.31) (*n* = 155)	9.660	0.008
Immunoglobulin–A	2.19(1.59–2.83) (*n* = 1505)	2.19(1.63–2.82) (*n* = 474)	2.19(1.54–2.81) (*n* = 876)	2.30(1.74–3.02) (*n* = 155)	4.350	0.114
Immunoglobulin–M	1.12(0.83–1.53) (*n* = 1506)	1.12(0.79–1.55) (*n* = 475)	1.13(0.83–1.52) (*n* = 876)	1.07(0.88–1.53) (*n* = 155)	0.341	0.843
Immunoglobulin–G	11.80(10.20–13.60) (*n* = 1508)	11.70(9.98–13.60) (*n* = 476)	11.70(10.10–13.50) (*n* = 877)	12.70(10.70–14.90) (*n* = 155)	15.225	0.000
Serum ferritin (μg/L)	120.70(49.30–242.90) (*n* = 1459)	140.70(57.98–253.63) (*n* = 450)	110.20(44.15–233.05) (*n* = 853)	126.70(59.33–250.79) (*n* = 156)	5.411	0.067
CD3	1151.09(806.87–1582.38) (*n* = 648)	1247.54(877.27–1712.52) (*n* = 247)	1071.82(730.31–1433.89) (*n* = 333)	1153.94(858.08–1576.71) (*n* = 66)	15.367	0.000
CD4	628.78(419.59–891.08) (*n* = 646)	697.00(505.06–942.87) (*n* = 247)	560.14(384.32–815.31) (*n* = 333)	646.87(470.32–911.36) (*n* = 66)	19.186	0.000
CD8	426.02(287.33–591.60) (*n* = 645)	464.48(300.99–663.13) (*n* = 245)	416.78(254.93–560.78) (*n* = 333)	458.73(312.61–584.30) (*n* = 67)	5.813	0.055
CD4/CD8	1.47(1.12–1.89) (*n* = 954)	1.47(1.17–1.97) (*n* = 305)	1.46(1.10–1.85) (*n* = 547)	1.52(1.07–1.97) (*n* = 102)	2.902	0.234
B cell	145.28(85.63–245.02) (*n* = 183)	196.97(40.24–314.40) (*n* = 46)	142.67(86.00–217.45) (*n* = 117)	163.45(86.54–279.07) (*n* = 20)	2.250	0.325
NK cell	160.10(81.48–230.76) (*n* = 180)	155.32(63.02–223.91) (*n* = 46)	146.60(80.82–232.10) (*n* = 115)	193.00(138.59–274.30) (*n* = 19)	2.501	0.286
Lactate dehydrogenase	200.00(173.00–235.00) (*n* = 1933)	197.00(170.00–231.00) (*n* = 648)	200.00(173.00–239.00) (*n* = 1105)	208.00(177.25–237.00) (*n* = 180)	5.918	0.052
Uric acid	331.0(265.00–404.00) (*n* = 1936)	336.00(268.75–407.00) (*n* = 650)	328.50(261.00–404.00) (*n* = 1106)	323.50(266.00–397.50) (*n* = 180)	1.576	0.455
Patch shadow	–	–	–	119(61.34%)	–	–
Ground glass opacity	–	–	–	103(53.09%)	–	–
Strip cord shadow	–	–	–	76(39.18%)	–	–
Nodular shadow	–	–	–	29(14.95%)	–	–

SARS-CoV-2, severe acute respiratory syndrome coronavirus 2; NK cell, natural kill cell; INR, international normalized ratio.

### Main treatment options

All patients received traditional Chinese medicine treatment. In addition, 33 (1.29%), 54 (2.13%), and 12 (0.47%) patients received conventional nasal cannula oxygenation, oral antibiotic therapy (e.g., moxifloxacin and cefdinir) due to the consideration of bacterial co-infections, and antiviral therapy with oseltamivir phosphate, respectively.

### Risk factors for delayed discharge or release from quarantine

To explore the risk factors for delayed discharge or release from quarantine, patients with incomplete laboratory results were excluded from the analysis. Subsequently, using the median length of hospital stay of 17 days as the threshold, patients were divided into the control (< 17 days, *n* = 1210) and observation (≥ 17 days, *n* = 1331) groups. Crude logistic regression analysis showed that sex, disease classification, as well as lymphocyte and platelet counts, were related to delays in discharge or release from quarantine. Multivariable logistic regression analysis showed that only the disease classification and lymphocyte count were risk factors for delayed discharge or release from quarantine (Table 3).

**Table 3:** Crude and adjusted logistic regression analyses of the risk factors for delayed discharge from the hospital.

Variables	Univariate analysis		Multivariate analysis	
	OR (95% CI)	*P*-value	OR (95% CI)	*P*-value
Clinical classification	1.500 (1.318–1.707)	0.000	1.378 (1.88–1.599)	0.000
Lymphocyte count	0.742 (0.670–0.824)	0.000	0.757 (0.684–0.839)	0.000
PLT	0.998 (0.997–1.000)	0.042	1.000 (0.998–1.001)	0.636
Gender	0.843 (0.721–0.985)	0.032	0.889 (0.742–1.066)	0.204

*CI*, confidence interval; *OR*, odds ratio; *PLT*, platelet count.

## Discussion

**Figure 2:**
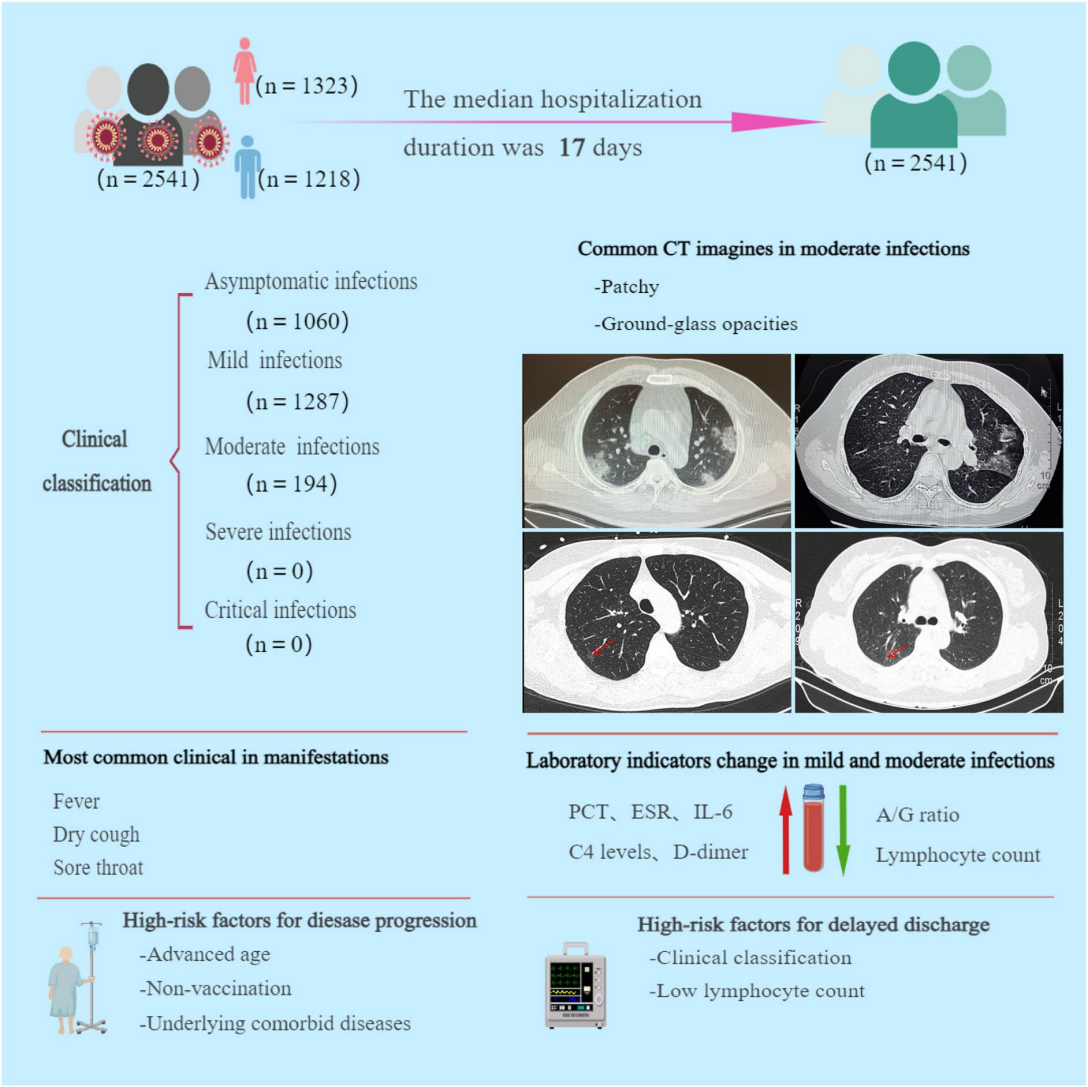
Graphical Abstract.

This retrospective clinical study involved patients infected with the community-transmitted SARS-CoV-2 Omicron BA.2 in China. Our results show that patients with SARS-CoV-2 Omicron BA.2 infection in Quanzhou were associated with lower median age, mild clinical presentation, shorter hospital stays, no severe cases and deaths, higher coverage of COVID-19 vaccine, and better prognosis compared with those infected with the original strain (Fig. 2). Despite the relatively low virulence of the Omicron variant, unvaccinated older adults, especially those with underlying comorbid diseases, were at high risk of suffering from both severe infection and death^9^. The predominance of asymptomatic infections in the vaccinated patients in this study and that of mild infections in the unvaccinated patients suggest that COVID-19 vaccination is protective against the Omicron variant, and the protective effect is independent of the number of vaccination doses.

Existing studies have suggested that women are less susceptible to SARS-CoV-2 due to the protective effects of the X chromosome and sex hormones^10^, with a lower risk of death in women infected with SARS-CoV-2 than in men (hazard ratio 1.59, 95% confidence interval: 1.53–1.65)^11^. In contrast, this study shows that the percentage of infected men was lower than that of infected women (47.93% vs. 52.07%). Moreover, the percentage of men with moderate infections was significantly lower than that of men with asymptomatic infections, suggesting that women are more susceptible to the Omicron variant BA.2 and more likely to have moderate infections. Unlike those infected with the original SARS-CoV-2 strain, the median age of patients infected with the Omicron variant was relatively low, with a downward shift in the age of infection^12–14^. Although patients aged ≥ 60 accounted for only 8.62% of the enrolled population, a higher percentage of them were with moderate infections than those aged < 18 and 18–59 years, suggesting that older age is still an important factor influencing disease progression. Another independent risk factor for disease progression is underlying comorbid disease^15^ rather than various mutations in the Omicron sublineage BA.2.2^16^. The role of underlying diseases in the progression of Omicron infections was further highlighted by the fact that 10.82% of the patients in our study had comorbidities, and the percentage of moderate infections was higher in this sub-population than in the rest of the patients without pre-existing conditions.

In this study, 41.72% of the patients infected with Omicron BA.2 were asymptomatic, and the clinical manifestations of confirmed cases were relatively mild. The most common symptoms of confirmed cases were fever, dry cough, sore throat, fatigue, and muscle aches. Meanwhile, anosmia and ageusia, conjunctivitis, nausea, and vomiting were relatively rare, consistent with the results reported in a South Korea-based study^17^. The relatively high percentage of asymptomatic infections poses a great challenge to effective prevention and control of the pandemic. Furthermore, nucleic acid testing and COVID-19 antigen detection remain important tools to help in dynamic monitoring of the pandemic and prompt identification of infected persons^18^. Although asymptomatic and mild infections do not lead to changes in pulmonary imaging, the imaging parameter plays an irreplaceable role in clinical diagnosis and staging. Similar to the original SARS-CoV-2 strain, infections with the Omicron variant can also damage the lungs. In our study, damage manifested as patchy and ground-glass opacities in computed tomography images, with most lesions distributed in the peripheral zone and the subpleural areas. The difference was that pulmonary lesions in patients infected with the Omicron variant were significantly smaller or nodular, consistent with that reported in an Italian study^19^. The main reason for this difference is that the Omicron variant has a reduced binding capacity to the TMPRSS2 protein, significantly reducing the amount of virus entering the lung cells^20,21^.

Moreover, laboratory tests play an integral role in monitoring the severity of the disease and its treatment. Current studies have shown that decreased lymphocyte and increased neutrophil counts are significantly and positively associated with mortality^22^. Although there were no fatalities in this study, it is apparent that lymphocyte count was significantly reduced in moderate and mild infections compared with that in asymptomatic infections, which is consistent with the results of a previous Chinese study^23^. Inflammatory indicators of infection, such as procalcitonin (PCT), ESR, IL-6, and D-dimer levels, are effective predictors of mortality in inpatients in intensive care units^24^. In this study, the PCT, ESR, IL-6, and D-dimer levels were progressively elevated during asymptomatic, mild, and moderate infections. In particular, the IL-6 level was significantly elevated, with 7.48% of infected patients having an IL-6 level of > 1,000 mg/L and 2.32% of infected patients surpassing the upper limit (> 5,000 mg/L). Meanwhile, the CRP level, ESR, and leukocyte count were not simultaneously elevated to a high level. This observation ruled out the possibility of bacterial infections and suggested that over-activation of the IL-6 signaling pathway by the Omicron variants may be involved. However, the specific mechanism needs further investigation. These results suggest that dynamic monitoring of routine blood parameters, especially lymphocyte count and inflammatory indicators of infection, is useful in predicting the severity of the disease.

A previous study showed that the incidence of liver dysfunction in patients with COVID-19 was approximately 14–53%^25^, which was significantly higher than that observed in this study (7.48%). Our results can potentially be attributed to milder infections and the Omicron variant. Although levels of ALB, GLB, ALT, GGT, and TBIL in most infected patients were within normal ranges, approximately 12.28% of these patients still developed hypo-albuminemia, a condition that can be used as a marker to assess the severity of injury to the endothelial cells of the pulmonary capillary in infected patients^26^. Low ALB levels and high GLB levels in moderate infections led to a further decrease in the A/G ratio, and thus monitoring the A/G ratio helps determine the prognosis of the disease. Serum ferritin and lactate dehydrogenase are considered predictors of disease severity and progression in SARS-CoV-2-infected patients^27^; however, neither was associated with disease severity in Omicron variant-infected patients in this study. Moreover, this study did not observe acute kidney injury induced by the interaction of SARS-CoV-2 nucleoproteins with Smad3 signaling molecules^28^.

Several studies have demonstrated the presence of complement system activation in patients with COVID-19, as evidenced by a decrease in both complement C3 and complement C4 levels, as well as a significant correlation between the decrease in disease severity and high mortality^29,30^. Complement C4 level dropped in only 2.94% of infected patients in this study but was progressively elevated in asymptomatic, mild, and moderate infections, suggesting that the increase in complement C4 level is associated with disease severity in Omicron variant-infected patients. Further, high complement C3 level is an independent risk factor for delayed hospital discharge in patients infected with the original SARS-CoV-2 strain^13^.

This study has some limitations. First, all investigated infections originated only from the Quanzhou area, and there were no severe cases; hence, the epidemiological and clinical features did not comprehensively cover all SARS-CoV-2 Omicron infections. Second, the incubation period, CT of RT-qPCR for nucleic acid testing, and duration of symptoms of infected patients were not investigated. Third, the effect of the traditional Chinese medicine decoction on disease outcomes was not clarified. Although traditional Chinese medicine can play a role in adjuvant therapy, it is not the main therapy. Traditional Chinese medicine has certain advantages in relieving symptoms, improving immunity, and promoting rehabilitation, which has been involved in the treatment of patients with COVID-19 enrolled in this research institute. In this study, all patients with COVID-19 were treated with traditional Chinese medicine decoction as Yiqi Jiedu prescription, and clarifying its effect was impossible without comparison to other treatments or controls.

Despite these limitations, this was a rare real-world study of a large number of COVID-19 community infections in coastal areas of China. The number of infected patients included was large, and all infected patients were hospitalized. The observation and judgment of the condition of infected people were supported by medical staff with professional knowledge. This is a comprehensive and objective study with a detailed description of the demography, clinical manifestations, imaging features of the lung, and clinical outcomes of infected patients, which would have a certain clinical reference value.

In conclusion, this study revealed that patients infected with the SARS-CoV-2 Omicron variant in Quanzhou had a high rate of novel coronavirus vaccination, mild clinical manifestations, short hospitalization duration, and good prognosis, with no severe and death-related cases. This study reveals, to a certain extent, the basic characteristics and prognostic outcomes of Omicron BA.2 infections. Our findings would provide a reference for the prognostic prediction and medical resource allocation regarding Omicron BA.2 infections.

## Data Availability

All relevant information is provided in this current manuscript. If required, the data presented in this work can be shared by e-mail. If you need the data, please contact Xueping Yu.
